# Effects of Chronic Static Stretching on Maximal Strength and Muscle Hypertrophy: A Systematic Review and Meta-Analysis with Meta-Regression

**DOI:** 10.1186/s40798-024-00706-8

**Published:** 2024-04-19

**Authors:** Konstantin Warneke, Lars Hubertus Lohmann, David G. Behm, Klaus Wirth, Michael Keiner, Stephan Schiemann, Jan Wilke

**Affiliations:** 1 Institute of Human Movement Science, Sport and Health, University of Graz, Graz, Austria; 2 Department of Human Motion Science and Exercise Physiology, Friedrich Schiller University, 07743 Jena, Germany; 3 School of Human Kinetics and Recreation, Newfoundland and Labrador, Memorial University of Newfoundland, St. John’s, Canada; 4 University of Applied Sciences Wiener Neustadt, Wiener Neustadt, Austria; 5 Department of Sport Science, German University of Health & Sport, Ismaning, Germany; 6 Institute of Exercise, Sport and Health, Leuphana University, Lüneburg, Germany; 7 Department of Movement Sciences, University of Klagenfurt, Klagenfurt am Wörthersee, Austria

**Keywords:** Stretching, Exercise, Maximum strength, Hypertrophy, Long-lasting

## Abstract

**Background:**

Increases in maximal strength and muscle volume represent central aims of training interventions. Recent research suggested that the chronic application of stretch may be effective in inducing hypertrophy. The present systematic review therefore aimed to syntheisize the evidence on changes of strength and muscle volume following chronic static stretching.

**Methods:**

Three data bases were sceened to conduct a systematic review with meta-analysis. Studies using randomized, controlled trials with longitudinal (≥ 2 weeks) design, investigating strength and muscle volume following static stretching in humans, were included. Study quality was rated by two examiners using the PEDro scale.

**Results:**

A total of 42 studies with 1318 cumulative participants were identified. Meta-analyses using robust variance estimation showed small stretch-mediated maximal strength increases (d = 0.30 *p* < 0.001) with stretching duration and intervention time as significant moderators. Including all studies, stretching induced small magnitude, but significant hypertrophy effects (d = 0.20). Longer stretching durations and intervention periods as well as higher training frequencies revealed small (d = 0.26–0.28), but significant effects (*p* < 0.001–0.005), while lower dosage did not reach the level of significance (*p* = 0.13–0.39).

**Conclusions:**

While of minor effectiveness, chronic static stretching represents a possible alternative to resistance training when aiming to improve strength and increase muscle size. As a dose-response relationship may exist, higher stretch durations and frequencies as well as long program durations should be further elaborated.

**Supplementary Information:**

The online version contains supplementary material available at 10.1186/s40798-024-00706-8.

## Key Points

**•** While animal research consistently showed chronic stretch-mediated hypertrophy and strength increases, literature in humans draws an inconclusive picture, possibly due to lack of comparability of stretching parameters, such as duration and frequency.
**•** Our systematic review is the first that included studies using comparable stretching durations of up to two hours in humans, which showed small magnitude maximal strength increases and muscle hypertrophy.
**•** Even though less effective, high volume stretching might provide a sufficient alternative to strength training when aiming to induce muscle hypertrophy and strength increases. It must be noted that comparatively high training effort is opposed by comparatively small adaptations, suggesting a preference for the more efficient strength training if applicable.

**Supplementary Information:**

The online version contains supplementary material available at 10.1186/s40798-024-00706-8.

## Background

Stretch training is commonly used to achieve improvements in flexibility [1, 2], with widespread applications in sports conditioning and orthopedic physical therapy [3, 4]. While it was widely accepted in the 1980s that static stretching should be included in warm-up routines [5–7], current evidence questions the implementation of (static) stretching during warm-up due to its detrimental impact on subsequent sports performance [8–10].

Despite adverse acute effects, static stretching may be beneficial for athletes if performed in the long-term [11, 12]. A recent systematic review with meta-analysis evaluating animal studies found chronic stretching of the anterior latissimus dorsi in chickens and quails (for up to 24 h per day, seven days per week) substantially increased muscle mass by up to 319% (d = 8.5) due to increases in muscle cross-sectional area (up to 142%; d = 7.9). Besides these structural changes, gains in maximal strength (up to 95%; d = 12.4) [13] were observed. Interestingly, investigations aiming to translate animals’ muscle adaptions to humans were requested as early as in 1983: “Thirty minutes of stretching per day is certainly within normal physiological limits, and as a result may be applied to human muscle with hopes that similar adaptations would occur” [14].

Stretching effects on hypertrophy [15, 16] and strength [17, 18] in humans were previously reviewed pointing out only small strength increases (under dynamic conditions [17]) while muscle hypertrophy was exclusively evident using high intensity stretching [16]. However, even though recent reviews were performed in 2023, they missed inclusion of new literature that – for the first time – applied static stretching with continuous stretching durations up to two hours [19–26], which might lead to an under- or overestimation of the current evidence.

Consequently, the aim of this systematic review with meta-analysis was to investigate changes in muscle size and maximum strength following chronic static stretching interventions in humans. We hypothesized that stretching programs, performed in the long-term, would lead to increases in both outcomes. Based on findings from animal research, we assumed that previous stretching volume was not sufficient. Therefore, we hypothesized longer stretching session durations and intervention periods, as well as high training frequencies would trigger improvements, while lower durations/frequencies would not elicit relevant changes.

## Methods

A systematic review and meta-analysis using robust variance estimation was performed adhering to the PRISMA (Preferred Reporting Items for Systematic Reviews and Meta-Analyses) guidelines. The study was registered in the PROSPERO database (CRD42023411225).

### Literature Search

Two independent investigators (KoW & LHL) conducted a systematic literature search using MEDLINE/PubMed, Web of Science and SPORTDiscus (March 2023) and updated in January 2024. The following inclusion criteria were applied: (1) randomized, controlled study design; (2) static stretching intervention with a duration of at least two weeks, performed in humans; (3) measurement of (a) maximal strength or related parameters such as active peak torque and/or (b) markers of muscle size (i.e., cross-sectional area, muscle thickness). Studies assessing acute effects, combining static stretch training with other (active) training protocols such as resistance training or neuromuscular facilitation, or including patients were excluded. The search terms (Online Supplemental Material) were created based on the requirements of each database. As an example, the terms for PubMed were as follows:

((stretch*) AND (performance OR strength OR 1RM OR force OR MVC OR (maxim* AND “voluntary contraction”) OR hypertrophy OR “muscle cross-sectional area” OR CSA OR “muscle thickness” OR “muscle mass” OR “muscle volume”) NOT (acute OR postural OR pnf OR “proprioceptive neuromuscular facilitation” OR “stretch shortening”)).

In addition to database searches, the reference lists of all included studies were screened for further eligible articles [27].

### Methodological Study Quality and Risk of Bias

The assessment of study quality was performed by two independent investigators (KW1 & LHL) using the PEDro scale for randomized, controlled trials [28, 29]. If consensus could not be reached, a third rater casting the decisive vote was consulted (MK). The PEDro scale (Table A in Supplemental Material) was used in previous reviews with meta-analysis on exercise and exercise therapy [30, 31].

Risk of publication bias was examined using visual inspection of funnel plots [32], which were created using the method of Fernandez-Castilla et al. [33]. Additionally, Egger’s regression tests incorporating robust variance estimation for funnel plot asymmetry were applied [34]. The certainty about the evidence was rated as very low, low, moderate or high using the criteria proposed by the GRADE working group [35]. Generally, the quality of evidence of randomized trials is considered high and thereafter adjusted within the GRADE framework. In case of limitations in study design or execution, inconsistency of results, indirectness of evidence, imprecision or publication bias, one point is subtracted for each weakness. Conversely, large-magnitude effects or a dose-response gradient each lead to addition of one point to the quality of evidence rating.

### Data Processing and Statistics

The means (M) and standard deviations (SD) from pre- and post-intervention tests were extracted for all parameters and study arms (stretching and inactive control). In case of missing data, the authors of the primary studies were contacted. Changes from pre to post were computed as M_(posttest)_ – M_(pretest)_ and standard deviations were pooled as

$${SD}_{pooled}=\sqrt{\frac{\left({n}_{1}-1\right)*{SD}_{1}^{2}+\left({n}_{2}-1\right)*{SD}_{2}^{2}}{\left({n}_{1}-1\right)+({n}_{2}-1)}}$$

To account for multiple within-study outcome dependency with unknown origin of covariances, meta-analytical calculation was performed using robust variance estimation [36]. Standardized mean differences (SMD) and 95% confidence intervals (CI) for maximal strength capacity and muscle size changes (including both muscle thickness and muscle cross-sectional area) were pooled from fitting parameters from all included studies. We used R (R Foundation for Statistical Computing, Vienna, Austria) with the robumeta, version 2.0 [36] and metapackages. Obtained effect sizes (ES) were interpreted as 0 ≤ d < 0.2 trivial, 0.2 ≤ d < 0.5 small, 0.5 ≤ d < 0.8 moderate, or d ≥ 0.8 large [37], while τ² was used to explore study outcome heterogeneity, with classifications equal to effect sizes.

Meta-regression was performed using the robumeta package for dependent study outcomes, as described by Fisher & Tipton [36]. Furthermore, to quantify the influence of quantifable outcome moderators (stretching duration, intervention period and training frequency) when aiming to enhance maximal strength and muscle size, sub-analyses were performed for three variables: intervention duration, session duration and exercise frequency. For moderating variables (duration, intervention period and training frequency), we used the median-split for cut-off determination (intervention duration: small: <6 weeks vs. high: ≥ 6 weeks, frequency: low: <5 sessions vs. high: ≥5 sessions, stretching duration: short: <15 min vs. long: ≥15 min. To test for significant differences in mean effect size of sub-groups, the Welsh test was performed due to violation of normal distribution. If several study effects were presented mean effects for each study were calculated to account for within-study dependency in effect size comparsions.

## Results

### Search Results

**Fig. 1:**
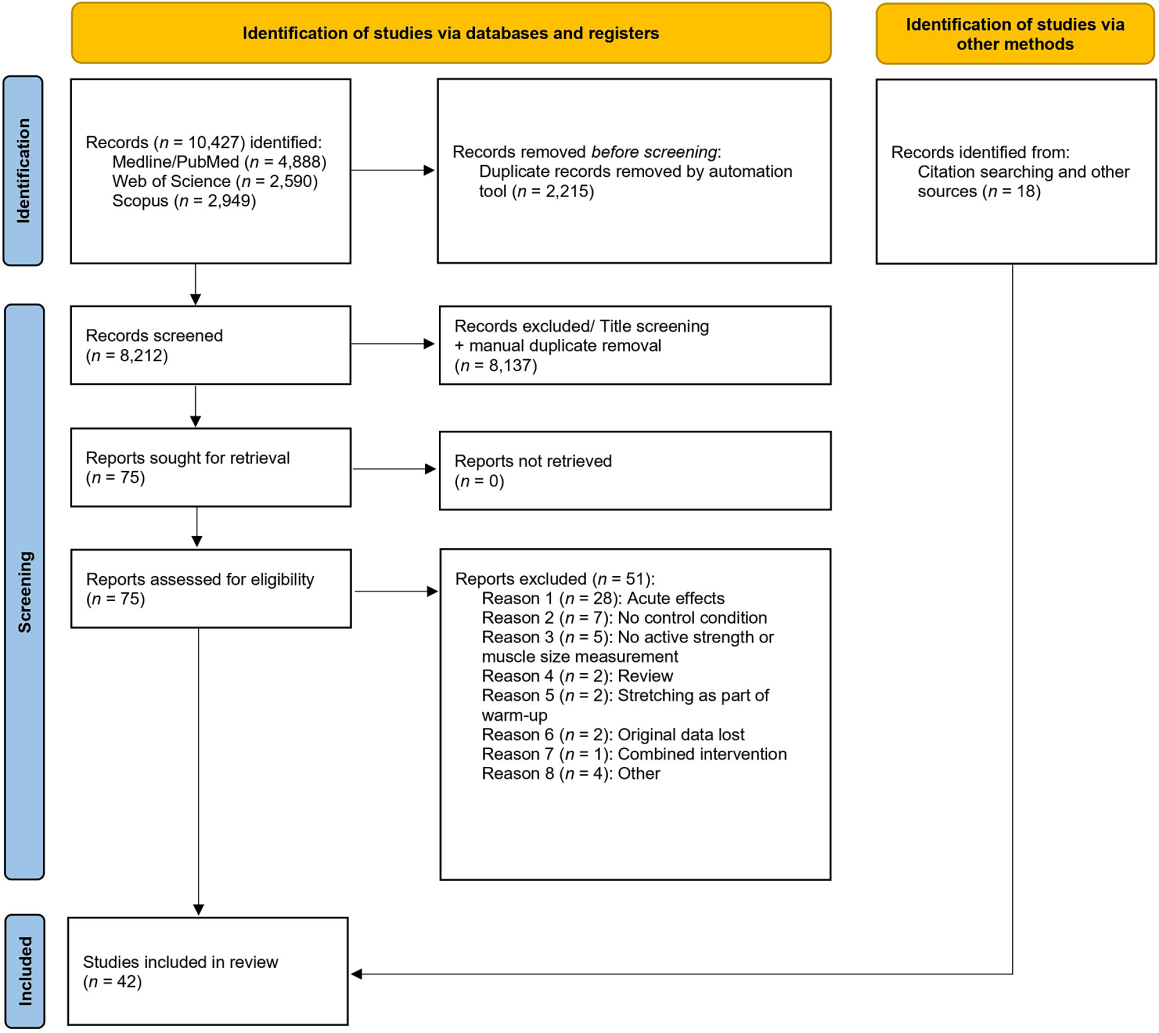
Flow chart of literature search

Figure 1 displays the flow of the literature search.

Collectively, the queries in the three databases returned 10,427 hits. After application of inclusion and exclusion criteria, a total of 42 eligible studies with 1318 participants were identified. Among these, 36 studies with 85 ES [19–26, 38–65] investigated strength parameters. Nineteen (19) studies [21–24, 26, 39, 66–68, 51, 52, 55, 57, 58, 69–71, 63, 65] with 45 ES examined markers of muscle size.

### Methodological Quality, Risk of Bias and Quality of Evidence

Per average, the methodological quality of the included studies was rated as fair [72] (mean 4.17 ± 1.4 out of 10 points; range 2 to 8 points; see Table A in Supplemental Material). For both outcomes (muscle volume and maximal strength), the quality of evidence was downgraded by 2 levels (high to low) due to high risk of bias (limitations in study quality: fair PEDro score and heterogeneity in study designs). In case of the sub-analyses for session and intervention duration (outcomes of maximal strength), the quality of evidence was upgraded by one level due to moderate to strong associations (low to moderate effect sizes, mostly on same side effect).

### Quantitative Synthesis

Table 1 provides the study characteristics of included articles, while Table 2 summarizes the quantitative analysis results for overall and different subgroups.

**Table 1:** Description of included studies

Article	Subjects	Muscle group	Intervention	Results (mean ± standard deviations)
Abdel-Aziem & Mohammad [38]	*N* = 75. IG1 (untrained group): *n* = 25. IG2 (trained group): *n* = 25. CG: *n* = 25.	Plantar flexors	Stretching was performed by staying 2 to 3 feet from a wall and moving the right foot from the wall until participant felt a stretching in the posterior chain of the calf muscle. The hands had to be placed against the wall, and extend the right hip and the right knee placing their hands against the wall and maintaining their right hip and knee in extension	*Maximal concentric torque in the Plantar flexion with 30°/s (in NM)* Pre-test IG1: 82.28 ± 15.46, Post-test: IG1 89.80 ± 14.31 Pre-test IG2: 80.16 ± 14.57, Post-test: IG2 80.04 ± 14.22 Pre-test CG: 78.76 ± 13.36, Post-test: CG 78.84 ± 13.16 *Maximal concentric torque in the Plantar flexion with 120°/s (in NM)* Pre-test IG1: 55.04 ± 10.77, Post-test IG1: 61.35 ± 9.78 Pre-test IG2: 50.96 ± 7.64, Post-test IG2: 58.42 ± 7.64 Pre-test CG: 49.75 ± 6.94, Post-test CG: 50.00 ± 6.94 *Maximal eccentric torque in the Plantar flexion with 30°/s (in NM)* Pre-test IG1: 85.61 ± 14.99, Post-test IG1: 95.81 ± 12.58 Pre-test IG2: 81.08 ± 12.98, Post-test IG2: 90.79 ± 14.03 Pre-test CG: 80.16 ± 13.61, Post-test CG: 81.04 ± 13.84 *Maximal eccentric torque in the Plantar flexion with 120°/s (in NM)* Pre-test IG1: 92.52 ± 10.74, Post-test IG1: 104.09 ± 10.45 Pre-test IG2: 88.47 ± 9.64, Post-test IG2: 96.84 ± 11.95 Pre-test CG: 87.58 ± 15.21, Post-test CG: 87.96 ± 15.17
Akagi & Takahashi [39]	*N* = 19, right and left leg	Plantar flexors	3 × 2 min stretching with one minute rest in between on 6 days per week for 5 weeks by using a stretching board, stretching was performed without pain and discomfort	*Muscle thickness in the plantar flexors (in mm)* Pre-test IG: 7.6 ± 0.8, Post-test IG: 7.6 ± 0.7 Pre-test CG: 7.6 ± 0.7, Post-test CG: 7.6 ± 0.7 *Maximal Joint torque (in Nm)* Pre-test IG: 118 ± 25, Post-test IG: 121 ± 20 Pre-test CG: 116 ± 21, Post-test CG: 119 ± 17
Andrade et al. [66]	*N* = 39. IG: *n* = 21. CG: *n* = 18.	Plantar flexors	2 static stretching exercises each performed 5 × 45 s per session, 5 sessions per week for 12 weeks.	*Muscle thickness in gastrocnemius medialis (in mm)* Pre-test IG: 1.6 ± 0.3, Post-test IG: 1.7 ± 0.2 Pre-test CG: 1.5 ± 0.2, Post-test IG: 1.6 ± 0.1 *Muscle thickness in gastrocnemius lateralis (in mm)* Pre-test IG: 1.3 ± 0.2, Post-test IG: 1.3 ± 0.2 Pre-test CG: 1.4 ± 0.2, Post-test IG: 1.3 ± 0.1
Barbosa et al. [40]	*N* = 45. IG1 (static stretching): *n* = 15. IG2 (dynamic stretching): *n* = 15. CG: *n* = 15.	Hamstrings	Stretching was performed 3 times per week until 10 sessions are completed, static stretching with 3 × 30 s, dynamic stretching 3 × 30 repetitions. Static stretching was performed from a supine position via hip flexion with extended knee joint until point of discomfort	*Maximal eccentric peak torque (in Nm/kg)* Pre-test IG 250.58 ± 37.54, Post-test IG 211.95 ± 46.45 Pre-test CG 238.71 ± 42.10, Post-test CG 229.43 ± 41.10
Brusco et al. [41]	*N* = 13. Contralateral leg as control condition.	Hamstrings	Stretching was performed with eight repetitions of 60 s each on two non-consecutive days per week for six weeks.	*Dynamic torque (in Nm)* Pre-test IG: 110.2 ± 19.4, Post-test IG: 106.3 ± 18.1 Pre-test CG: 107.2 ± 11.4, Post-test CG: 100.1 ± 20.6
Caldwell et al. [42]	*N* = 30. IG1 (stretching 1x /day): *n* = 10. IG2 (stretching 2x /day): *n* = 10. CG: *n* = 10.	Hamstrings & Quadriceps	3 × 30 s stretching training with 15 s rest between each set for 2 weeks. Stretching was performed daily or twice daily	*Maximal strength in the hamstrings* Pre-test IG 1x per day: 109.1 ± 11.9, Post-test IG 1x per day: 118.5 ± 12.9 Pre-test IG 2x per day: 110.5 ± 9.6, Post-test IG 2x per day: 111.5 ± 13.1 Pre-test CG: 110.8 ± 17.4, Post-test CG: 110.5 ± 17.6 *Maximal strength in the Quadriceps* Pre-test IG 1x per day: 438.7 ± 91.8, Post-test IG 1x per day: 445.2 ± 78.4 Pre-test IG 2x per day: 546.5 ± 46.9, Post-test IG 2x per day: 585.3 ± 48.3 Pre-test CG: 492.9 ± 99.4, Post-test CG: 472.5 ± 87.2
Chen et al. [43]	*N* = 30, three groups, CG, *n* = 10, stretching group, *n* = 10, PNF group, *n* = 10	Hamstrings	Unilateral hamstring stretch with support of the investigator to reach point of discomfort. Stretching was performed 30 × 30 s 3x per week for 8 weeks	*MVC in the knee flexion (in Nm)* Pre-test IG 72.7 ± 3.2, Post-test IG 79.0 ± 3.0 Pre-test CG 70.4 ± 3.9, Post-test CG 71.0 ± 4.0 *MVC in the knee extension (in Nm)* Pre-test IG 124.9 ± 7.8, Post-test IG 128.7 ± 8.3 Pre-test CG 117.1 ± 6.7, Post-test CG 119.3 ± 6.8
Cini et al. [44]	*N* = 12. IG: *n* = 6. CG: *n* = 6.	Hamstrings	1 × 30 s hamstring stretch per session, three sessions per week for 4 weeks.	*Knee flexion peak torque (in Nm)* Pre-test IG: 66 ± 14.4, Post-test IG: 70.2 ± 8.4 Pre-test CG: 70.2 ± 12, Post-test CG: 73.2 ± 15
Freitas & Mil-Homens [67]	*N* = 10, IG *n* = 5, CG *n* = 5	Hamstrings	1 × 450 s of continuous stretching with highest tolerable torque with was ensured by increasing ROM every 90 s to new maximal ROM. Stretching was performed 5 days per week for 8 weeks.	*Muscle thickness of biceps femoris long head (in mm)* Pre-test IG: 145.4 ± 6.9, Post-test IG: 142.9 ± 8.8 Pre-test CG: 143.4 ± 15.2, Post-test CG: 141.9 ± 15.5
Ikeda & Ryushi [45],	*N* = 25. IG: *n* = 12. CG: *n* = 13	Knee extensors	6 × 30 s stretching of the quadriceps/knee extensors with a rest of 60 s in between 3 days per week for 6 weeks	*Maximal strength (in N)* Pre-test IG 4182 ± 958, Post-test IG 4607 ± 1015 Pre-test CG 3732 ± 714, Post-test CG 3725 ± 754
Kay et al. [68]	*N* = 26. IG: *n* = 13. CG: *n* = 13.	Knee extensors	5 sets of 12 × 3 s stretch on maximally contracted knee flexors induced by isokinetic dynamometer. 2 sessions per week for 6 weeks.	*Vastus lateralis thickness (in mm)* Pre-test IG: 27.3 ± 1.1, Post-test IG: 29.3 ± 1.1 Pre-test CG: 26.4 ± 1.3, Post-test CG: 26.4 ± 1.3
Kokkonen et al. [46]	*N* = 38. IG: *n* = 19. CG: *n* = 19.	Lower extremities	Stretching was performed 40 min per session 3x per week for 10 weeks including 15 exercises for the major muscle groups of the lower extremity. Each exercise was performed 3 × 15 s	*1RM in the knee flexion (in kg)* Pre-test IG 44.7 ± 14.5, Post-test IG 51.0 ± 14.1 Pre-test CG 46.1 ± 15.1, Post-test CG 47.0 ± 14.4 *1RM in the Knee extension (in kg)* Pre-test IG 63.8 ± 24.5, Post-test IG 82.0 ± 25.8 Pre-test CG 69.7 ± 21.5, Post-test CG 71.0 ± 20.8
Konrad & Tilp [47],	*N* = 41, IG: *n* = 21. CG: *n* = 20.	Plantar flexors	Stretching both plantar flexors unilateral in a standing wall push position until point of discomfort. Stretching was performed five times per week for six weeks with four times 30 s each session. The stretching was done alternating both legs with no rest in-between.	*Maximal voluntary contraction torque (in Nm)* Pre-test IG: 96.88 ± 35.8, Post-test IG: 100.4 ± 39.6 Pre-test CG: 92.7 ± 29.3, Post-test CG: 90.1 ± 33.2
Kubo et al. [48]	*N* = 8. Contralateral leg as control condition.	Plantar flexors	5 × 45 s unilateral static stretch, 2x per day for 20 consecutive days.	*Maximal voluntary contraction torque (in Nm)* Pre-test IG: 131 ± 17, Post-test IG: 132 ± 20 Pre-test CG: 130 ± 19, Post-test CG: 128 ± 18
LaRoche et al. [49]	*N* = 19. IG: *n* = 9. CG: *n* = 10.	Hamstrings	10 × 30 s static stretch per session, 3 sessions per week for 4 weeks.	*Knee flexion peak torque (in Nm)* Pre-test IG: 239.5 ± 49, Post-test IG: 257.5 ± 58.2 Pre-test CG: 278.8 ± 68.2, Post-test CG: 289.5 ± 55.4
Leslie et al. [50]	*N* = 16. IG: *n* = 8. CG: *n* = 8.	Hamstrings	Unilateral static stretching of both legs, 15 min per session, 3 sessions per week for 4 weeks.	*Knee flexion eccentric peak torque (in Nm)* Pre-test IG: 107.3 ± 28.4, Post-test IG: 113.3 ± 34.6 Pre-test CG: 99.5 ± 30.2, Post-test CG: 100.6 ± 30.5 *Knee flexion isometric peak torque (in Nm)* Pre-test IG: 93.4 ± 23.6, Post-test IG: 95 ± 30.1 Pre-test CG: 81.6 ± 21.9, Post-test CG: 84.6 ± 22 *Knee flexion concentric peak torque (in Nm)* Pre-test IG: 87.9 ± 21.8, Post-test IG: 92.6 ± 28.1 Pre-test CG: 81.5 ± 25.2, Post-test CG: 80 ± 22.9
Lima et al. [51]	*N* = 24. IG: *n* = 12. CG: *n* = 12.	Knee flexors & extensors	Unilateral 3 × 30 s static stretch per session for knee flexors and extensors each, 3 sessions per week for 8 weeks.	*Knee extension isometric peak torque (in Nm)* Pre-test IG: 218.1 ± 47.23, Post-test IG: 218.33 ± 40.80 Pre-test CG: 204.9 ± 4.79, Post-test CG: 211.2 ± 27.17 *Knee flexion isometric peak torque (in Nm)* Pre-test IG: 114.66 ± 27.18, Post-test IG: 122.25 ± 21.82 Pre-test CG: 120.54 ± 22.08, Post-test CG: 117.09 ± 22.42 *Vastus lateralis muscle thickness (in mm)* Pre-test IG:27.98 ± 6.67, Post-test IG: 26.192 ± 3.97 Pre-test CG: 24.58 ± 4.36, Post-test CG: 23.77 ± 3.97 *Biceps femoris muscle thickness (in mm)* Pre-test IG: 25.32 ± 4.87, Post-test IG: 25.99 ± 3.33 Pre-test CG: 22.65 ± 4.01, Post-test CG: 23.46 ± 3.55
Longo et al. [52]	*N* = 30, IG *n* = 15 CG *n* = 15	Plantar flexors	Stretching was performed 5 × 45 s with a rest of 15 s by using two exercises, 5x per week for 6 and 12 weeks, 450 s stretching duration per session. For the first exercise, a stretching board was used, for the second exercise a gymnastic band was used for stretching the plantar flexors with flexed hip and extended knee joint. Stretching was performed to the maximal point of discomfort	*Maximal strength in the plantar flexion (in Nm)* Pre-test IG 147.2 ± 32.1 6w Post-test IG 148.7 ± 32.4 12w Post-test IG 150.4 ± 32.6 Pre-test CG 151.7 ± 33.7 6w Post-test CG 152.8 ± 32.8 12w Post-test CG 153.9 ± 38.1 *Muscle thickness in the medial head of the gastrocnemius (in mm)* Pre-test IG 20.11 ± 2.38 6w Post-test IG 20.52 ± 2.55 12w Post-test IG 20.08 ± 1.80 Pre-test CG19.33 ± 2.46 6w Post-test CG 19.21 ± 2.13 12w Post-test CG 19.20 ± 2.24 *Muscle thickness in the lateral head of the gastrocnemius (in mm)* Pre-test IG 16.11 ± 2.65 6w Post-test IG 16.20 ± 2.99 12w Post-test IG 17.05 ± 2.32 Pre-test CG 15.07 ± 2.63 6w Post-test CG 15.13 ± 2.69 12w Post-test CG 15.06 ± 2.55 *Muscle thickness in the soleus (in mm)* Pre-test IG 15.17 ± 2.78 6w Post-test IG 15.45 ± 3.03 12w Post-test IG 15.28 ± 2.93 Pre-test CG 14.77 ± 3.89 6w Post-test CG 15.06 ± 4.19 12w Post-test CG 14.76 ± 4.22
Marshall et al. [53]	*N* = 22 IG = 11, CG = 11	Hamstrings	4 passive stretching exercises for the hamstrings were performed 5 times per week for 4 weeks. 1 session per week was supervised. Each stretch was performed 3 × 30 s.	*Hamstring strength 30°s* ^*− 1*^ *in Nm* *Pre-test IG 49.7 ± 16.2, Post-test IG 50.8 ± 20.2* *Pre-test CG 42.6 ± 10.8, Post-test CG 46.1* ± 13.9 Hamstring strength 120°s^− 1^ in Nm Pre-test IG 43.5 ± 12.8, Post-test 46.1 ± 12.1 Pre-test CG 48.7 ± 15.1, Post-test 49.3 ± 17.1
Minshull et al. [54]	*N* = 9. Contralateral leg as control condition.	Knee flexors	4 × 10 s stretch per sessoion, 3 sessions per week for 8 weeks.	*Knee flexion peak force (in N)* Pre-test IG: 329 ± 77, Post-test IG: 325 ± 75 Pre-test CG: 321 ± 64, Post-test CG: 317 ± 69
Mizuno [55]	*N* = 20, IG = 11, CG = 9	Plantar flexors	SS + ES = 4 sets of 30 s stretch with 30 s rest in-between sets, 3 times per week for 8 weeks, electrical stimulation with 80 Hz. Intensity with maximal mA without pain Calf muscle stretch performed with a stretching board. Weekly volume: 6 min. SS = only 4 × 30 s of static stretching with a stretching board, 3 times per week for 8 weeks.	*Maximal strength in the plantar flexion (in N)* Pre-test IG 454 ± 198, Post-test IG 562 ± 259 Pre-test IG (SS) 460 ± 104, Post-test IG (SS) 537 ± 110 Pre-test CG 465 ± 152, Post-test CG 485 ± 153
Morton et al. [56]	*N* = 24. IG: *n* = 12. CG: *n* = 12.	Full body	Participants stretched the pectoralis, deltoid, gluteus, adductors, hamstrings and quadriceps. Stretching was performed three times per week for five weeks. Total stretch time was 510 s per session.	*Knee extension peak torque at 180°* ^*−1*^ *(in Nm)* Pre-test IG: 88.38 ± 23.30, Post-test IG: 91.36 ± 25.21 Pre-test CG: 98.66 ± 24.51, Post-test CG: 95.86 ± 25.38 *Knee flexion peak torque at 180°* ^*−1*^ *(in Nm)* Pre-test IG: 44.29 ± 12.17, Post-test IG: 45.10 ± 11.06 Pre-test CG: 48.21 ± 10.89, Post-test CG: 50.46 ± 13.01
Moltubakk et al. [57]	*N* = 26	Plantar flexors	24 weeks, daily stretching 4 × 60 s, self administered with straight and bent knee joint by using a visual analog scale (VAS-scale)	*Muscle thickness in the medial head of the gastrocnemius (in mm)* Pre-test IG 19.7 ± 2.1, Post-test IG21.2 ± 2.0 Pre-test CG 19.8 ± 2.3, Post-test CG 21.7 ± 2.7
Nakamura et al. [58]	*N* = 40, IG1 high intensity stretching *n* = 14, IG2 low intensity *n* = 13, CG *n* = 13	Plantar flexors	3 × 60 s stretch of the plantar flexors for 4 weeks 3x per week using a stretching board, intensity was documented via 11-point verbal numerical scale, 0 = no pain at all, 10 = very, very painful	*Maximal isometric strength in the plantar flexors at 30° plantar flexion (in Nm)* Pre-test IG1 52.5 ± 20.1, Post-test IG1 55.9 ± 17.6 Pre-test IG2 54.8 ± 50.4, Post-test IG2 50.4 ± 20.0 Pre-test CG 61.4 ± 15.9, Post-test CG 64.1 ± 16.3 *Maximal isometric strength in the plantar flexors at neutral position (in Nm)* Pre-test IG1 146.9 ± 30.2, Post-test IG1 148.1 ± 22.0 Pre-test IG2 146.6 ± 27.1, Post-testIG2 148.8 ± 28.9 Pre-test CG 170.8 ± 24.0, Post-test CG 171.1 ± 19.4 *Maximal isometric strength in the plantar flexors at 15° dorsiflexion (in Nm)* Pre-test IG1 193.2 ± 43.6, Post-test IG1 198.4 ± 29.2 Pre-test IG2 191.0 ± 37.6, Post-test IG2 189.1 ± 42.9 Pre-test CG 191.4 ± 40.7, Post-test CG 193.5 ± 45 *Maximal dynamic strength with 30°/s (in Nm)* Pre-test IG1 115.2 ± 32.2, Post-test IG1 120.4 ± 21.9 Pre-test IG2 116.8 ± 22.8, Post-test IG2 120.0 ± 26.4 Pre-test CG 131.7 ± 17.3, Post-test CG 135.5 ± 15.2 *Maximal dynamic strength with 120°/s (in Nm)* Pre-test IG1 73.6 ± 16.2, Post-test IG1 74.5 ± 15.0 Pre-test IG2 74.2 ± 17.6, Post-test IG2 72.7 ± 20.0 Pre-test CG 68.2 ± 18.3, Post-test CG 69.7 ± 18.5 *Muscle thickness gastrocnemius medialis in mm* Pre-test IG1 19.2±2.9, Post-test IG1 19.5±2.5 Pre-test IG2 20.7±2.5, Post-test IG2 20.5±2.8 Pre-test CG 19.7±3.0, Post-test CG 19.4±2.7 *Muscle thickness gastrocnemius lateralis in mm* Pre-test IG1 15.8±2.4, Post-test IG1 15.5±2.4 Pre-test IG2 18.3±1.8, Post-test IG2 17.5±2.1 Pre-test CG 17.5±2.6, Post-test CG 17.5±2.5 *Muscle thickness soleus in mm* Pre-test IG1 17.7±3.1, Post-test IG1 17.4±3.3 Pre-test IG2 19.4±2.8, Post-test IG2 19.4±3.1 Pre-test CG 19.6±3.4, Post-test CG 19.7±2.6
Nakao et al. [59]	*N* = 30, IG = 15 CG = 15	Hamstrings	5 min stretching of the hamstring muscle 3x per week for 4 weeks, knee was extended passively until the point before discomfort	*Isokinetic strength 60°/s (in Nm)* Pre-test IG 77.7 ± 15.3, Post-test IG 85.9 ± 18.8 Pre-test CG 73.4 ± 17.6, Post-test CG 74.2 ± 14.2 *Isokinetic strength 180°/s (in Nm)* Pre-test IG 58.8 ± 15.8, Post-test IG 66.8 ± 14.6 Pre-test CG 58.6 ± 16, Post-test CG 59.6 ± 11.7 *Isometric strength (in Nm)* Pre-test IG 106.0 ± 22.1, Post-test IG 103.2 ± 19.8 Prestest CG 95.9 ± 20.2, Post-test CG 95.5 ± 16.1
Nelson et al. [60]	*N* = 25, IG = 13, CG = 12	Plantar flexors	10 weeks static stretching of the plantar flexors, 4 × 30 s stretching with 30 s rest by staying with the ball of the foot on a beam and let the beam hanging unsupported over the edge of the beam, participant should place the bodyweight on the right leg to improve stretching stimulus	Maximal strength measurement (in N) Pre-test IG 356 ± 76, Post-test IG 456 ± 85 Pre-test CG 369 ± 51, Post-test CG 368 ± 52
Nobrega et al. [61]	*N* = 21. IG: *n* = 11. CG: *n* = 10.	Full body	40 min stretching sessions, 2 sessions per week for 12 weeks.	*Right handgrip strength (in kg)* Pre-test IG: 33.7 ± 3.3, Post-test IG: 35.5 ± 3.2 Pre-test CG: 43.1 ± 3.9, Post-test CG: 42.6 ± 3.9 *Left handgrip strength (in kg)* Pre-test IG: 34.1 ± 3.1, Post-test IG: 35.9 ± 2.9 Pre-test CG: 37.8 ± 3.2, Post-test CG: 38.6 ± 3.3 *1RM Benchpress (in kg)* Pre-test IG: 30.2 ± 5.2, Post-test IG: 31.1 ± 5.3 Pre-test CG: 37 ± 6.5, Post-test CG: 34 ± 5.7 *1RM Leg press (in kg)* Pre-test IG: 85.6 ± 10.5, Post-test IG: 111.3 ± 14.6 Pre-test CG: 94.8 ± 8.2, Post-test CG: 105.3 ± 10.6
Panidi et al. [69]	*N* = 12. Contralateral leg as control condition.	Plantar flexors	Female volleyball athletes performed five stretch sessions per weeks for 12 weeks. Stretch sessions consisted of two sets of six static plantar flexor stretches using a stretching board. Each repetition lasted 45 s in week 1. Stretching time per repetition was increased by 15 s every 3 weeks, apart from the last 3 weeks. Total stretching duration thus increased from 540 s to 900 s.	*Gastrocnemius anatomical cross-sectional area (in cm* ^*2*^ *)* *Gastrocnemius medialis distal* Pre-test IG: 1.014 ± 0.235, Pos-test IG: 1.246 ± 0.293 Pre-test CG:0.97 ± 0.24, Post-test CG: 1.123 ± 0.261 *Gastrocnemius medialis medial* Pre-test IG: 1.828 ± 0.224, Post-test IG: 1.893 ± 0.170 Pre-test CG: 1.744 ± 0.210, Post-test CG: 1.796 ± 0.213 *Gastrocnemius lateralis distal* Pre-test IG: 0.792 ± 0.106, Post-test IG: 0.812 ± 0.195 Pretest CG: 0.836 ± 0.206, Post-test CG: 0.769 ± 0.15 *Gastrocnemius lateralis medial* Pre-test IG: 1.372 ± 0.23, Post-test IG: 1.394 ± 0.174 Pre-test CG: 1.396 ± 0.226, Post-test CG: 1.378 ± 0.206
Peixinho et al. [70]	*N* = 20. IG: *n* = 12. CG: *n* = 8.	Plantar flexors	Unilateral, alternating stretch using 2 stretch exercises each 2 × 30 s per session. 4–5 sessions per week for 10 weeks.	*Gastrocnemius cross-sectional area (in mm* ^*2*^ *)* Pre-test IG: 51.71 ± 11.02, Post-test IG: 52.3 ± 13.79 Pre-test CG: 50.94, Post-test CG: 52.21 ± 13.7
Reiner et al. [62]	*N* = 38, IG *n* = 19, CG *n* = 19	Pectoralis muscle	7 week pectoralis muscle stretching with 3 exercises performed for 5 min each	*Maximal voluntary contraction in long muscle length in N* Pre-test IG 288.9±118.4, Post-test IG 332.4±117.0 Pre-test CG 267.5±97.3, Post-test CG 258.5±100.3 *Maximal voluntary contraction in short muscle length in N* Pre-test IG 339.3±127.0, Post-test IG 365.6±124.7 Pretest CG 320.0±114.4, Post-test CG 301.8±118.0
Sekir et al. [71]	*N* = 34, static stretching *n* = 12, dynamic stretching *n* = 11, control *n* = 11	Peroneus longus, tibialis anterior	6 weeks stretching on 5 days per week for 4 × 30s, 2 muscles	*Muscle thickness in the peroneus in mm* IG Pre-test 20.9±3.9, Post-test 19.6±2.4 CG Pre-test 22.9±4.4, Post-test 22.9±3.6 *Muscle thickness in the tibialis anterior in mm* Pre-test IG 30.1±2.4, Post-test IG29.6±2.9 Pre-test CG 30.6±3.9, Post-test CG 30.6±3.0
Simpson et al. [63]	*N* = 21, IG = 11, CG = 10	Plantar flexors	6 weeks of stretching intervention IG = Stretching on self-determined non-dominant leg. Organised into blocks of five days with 2 days of stretching one day rest. Using leg press machine for calf stretch. 3 min of stretch 5 times per week with load of 20% 1RM with a weekly increase of 5%.	*Maximal strength in the plantar flexion (in Nm)* Pre-test IG 115.27 ± 18.49, Post-test IG 114.04 ± 11.36 Pre-test CG 104.21 ± 15.92, Post-test CG 111.72 ± 14.31 *Muscle thickness in cm* 5 days Pre-test IG 1.7±0.05, Post-test IG 1.8±0.05 Pre-test CG 1.6±0.05, Post-test CG 1.68±0.05 21 days Pre-test IG 1.7±0.05, Post-test IG 1.89±0.05 Pre-test CG 1.6±0.05, Post-test CG 1.7±0.05 42 days Pre-test IG 1.7±0.05, Post-test IG 1.84±0.05 Pre-test CG 1.6±0.05, Post-test CG 1.73±0.05
Warneke et al. [26]	*N* = 52, IG *n* = 27, CG *n* = 25	Plantar flexors	Daily stretching training for 1 × 60 min using a calf muscle stretching device to induce stretching or 6 weeks. Stretching was performed with an intensity of 9–10 on a pain scale from 1–10	*Maximal isometric strength in the plantar flexion with extended knee joint (in N)* Pre-test IG 1478.4 ± 309.7, Post-test IG 1726.00 ± 315.8 Pre-test CG 1585.4 ± 215.1, Post-test CG 1559.00 ± 217.8 *1RM testing in the plantar flexion with extended knee joint (in kg)* Pre-test IG 91.9 ± 35, Post-test IG 115.0 ± 32.2 Pre-test CG 96.9 ± 27.6, Post-test CG 95.0 ± 28.6 *Muscle thickness (in mm)* Pre-test IG 14.31 ± 2.42, Post-test IG 16.5 ± 2.78 Pre-test CG 14.54 ± 2.32, Post-test CG 14.85 ± 2.08
Warneke et al. [19]	*N* = 70, IG1 = 25 IG2 = 15 CG = 30	Plantar flexors	IG1 and IG2 performed a daily stretching program for six weeks. IG1 performed one hour stretching per day, IG2 performed two hours stretching per day Both groups used a calf muscle stretching orthosis	*Maximal isometric strength (in N)* Pre-test IG1 1195.3 ± 321.09, Post-test IG1 1364.54 ± 355.43 Pre-test IG2 1144.2 ± 244.7, Post-test IG2 1397.9 ± 366.5 Pre-test CG 1076.3 ± 364.5, Post-test CG 1056.0 ± 332.7
Warneke et al. [25]	*N* = 35, IG *n* = 17, CG *n* = 18	Plantar flexors	The IG performed a daily 10-minutes static stretching program using a calf muscle stretching board. The intervention was performed for 6 weeks	*Maximal isometric strength (in N)* Pre-test IG 2672.8 ± 688, Post-test IG 2918.7 ± 953.7 Pre-test CG 2204.4 ± 920.5, Post-test CG 2192.7 ± 953.7
Warneke et al. [24]	*N* = 46, IG *n* = 23, CG *n* = 23	Plantar flexors	Daily stretching training for 6 × 10 min using a calf muscle stretching device to induce stretching or 6 weeks. Stretching was performed with a intensity of 9–10 on a pain scale from 1–10 Orthosis was re-adjusted after 10 min of stretching to improve stretch intensity	*Maximal isometric strength in the plantar flexion with extended knee joint (in N)* Pre-test IG 1697.67 ± 389.76, Post-test IG 1856.81 ± 431.28 Pre-test CG 1623.86 ± 251.52, Post-test CG 1645.00 ± 275.00 *Maximal isometric strength in the plantar flexion with bent knee joint (in N)* Pre-test IG 1507.19 ± 333.16, Post-test IG 1580.29 ± 364.56 Pre-test CG 1413.10 ± 273.79, Post-test CG 1415.29 ± 266.23 *Muscle cross sectional area in the lateral head of the gastrocnemius (in mm²)* Pre-test IG 1015.33 ± 269.78, Post-test IG 1095.87 ± 275.74 Pre-test CG 1002.06 ± 216.72, Post-test CG 1022.06 ± 236.02 *Muscle cross sectional area in the medial head of the gastrocnemius (in mm²)* Pre-test IG 1715.54 ± 529.18, Post-test IG 1803.00 ± 535.64 Pre-test CG 1617.41 ± 428.08, Post-test CG 1630.35 ± 417.95 *Muscle thickness in the lateral head of the gastrocnemius (in mm)* Pre-test IG 14.58 ± 3.17, Post-test IG 15.54 ± 2.77 Pre-test CG 14.25 ± 2.52, Post-test CG 14.36 ± 2.53 *Muscle thickness in the medial head of the gastrocnemius (in mm)* Pre-test IG 18.43 ± 3.31, Post-test IG 19.66 ± 3.15 Pre-test CG 17.64 ± 3.29, Post-test CG 17.9 ± 3.28
Warneke et al. [23]	*N* = 69, stretching group *n* = 23, strength training group *n* = 23, CG *n* = 23	Plantar flexors	Daily stretching training for 1 × 60 min using a calf muscle stretching device to induce stretching or 6 weeks. Stretching was performed with a intensity of 9–10 on a pain scale from 1–10 Stretch training was compared to an commonly used resistance training routine for the plantar flexors	*Maximal isometric strength in the plantar flexion with extended knee joint (in N)* Pre-test IG 1522.61 ± 310.25, Post-test IG 1796.78 ± 368.08 Pre-test CG 1557.05 ± 284.46, Post-test CG 1607.80 ± 361.11 *Maximal isometric strength in the plantar flexion with bent knee joint (in N)* Pre-test IG 1314.7 ± 305.79, Post-test IG 1440.61 ± 332.67 Pre-test CG 1334.76 ± 235.36, Post-test CG1340.33 ± 205.81 *Muscle thickness in the lateral head of the gastrocnemius (in mm)* Pre-test IH 14.53 ± 2.43, Post-test IG 15.21 ± 2.11 Pre-test CG 14.33 ± 2.48, Post-test CG 14.40 ± 2.32 *Muscle thickness in the medial head of the gastrocnemius (in mm)* Pre-test IG 19.55 ± 2.59, Post-test IG 21.06 ± 2.88 Pre-test CG 18.49 ± 3.13, Post-test CG 18.41 ± 2.87
Warneke et al. [22]	*N* = 31, IG *n* = 18, CG *n* = 13	Pectoralis muscle	Pectoralis stretching on 4 days per week with 3 exercises each performed for 5 min for 8 weeks	*Isometric maximal strength in N* Pre-test IG 649.99±337.07, Post-test 685.53±325.11 Pre-test CG 600.5±251.37, Post-test CG 643.61±241.67 *1RM maximal strength (in kg)* Pre-test IG 75.35±33.62, Post-test IG 79.69±34.0 Pre-test CG 68.65±25.76, Post-test CG 69.19±26.11
Wilson et al. [64]	*N* = 32, CG *n* = 12, Immobilization group *n* = 10, Immobilization and stretching group *n* = 10	Plantar flexors	IG and Immobilization group had to use an immobilizator as long as as possible each day for 2 weeks. The stretching group had to perform a stretching routine for 10 × 30 s twice a day	*Isokinetic plantar flexion peak torque (in %BW)* Pre-test IG 32.7 ± 7.2, Post-test IG 36.6 ± 8.5 Pre-test CG 34.83 ± 6.66, Post-test CG 32.92 ± 7.86 Pre-test Immobilization 37.1 ± 11.00 Post-test Immobilization 31.20 ± 6.66
Wohlann et al. [21]	*N* = 44, IG *n* = 22, CG *n* = 22	Hamstrings, quadriceps, plantar flexors	Stretching intervention consisted of 4 exercises for the lower extremity. Stretching was performed daily 5 min per exercise for 6 weeks, high stretching intensity was performed by using a stretching pain scale from 1–10, stretching should be set to 9–10.	*Maximal strength in the leg press (in N)* Pre-test IG 789.1 ± 173.6, Post-test IG 823.8 ± 190.5 Pre-test CG 816.1 ± 168, Post-test CG 817.8 ± 179.9 *Muscle thickness in mm* Pre-test IG 154.5±26.3, Post-test IG 164.8±27.7 Pre-test CG 163.4±24.4, Post-test CG 165.3±23.2
Wohlann et al. [20]	*N* = 81, stretching group *n* = 27, strength group *n* = 27, CG *n* = 27	Pectoralis muscle	Supervised pectoralis stretch for 8 weeks, continuous for 15 min, 4 days per week	*Isometric maximal strength in N* Pre-test IG 461.3±196.6, Post-test IG 508.1±207.1 Pre-test CG 275.5±180.1, Post-test IG 479.1±179.4 *Muscle thickness pectoralis left* Pre-test IG 25.7±7.3, Post-test IG: 27.4±7.4 Pre-test CG 27.3±6.6, Post-test IG 27.2±6.4 *Muscle thickness pectoralis right in mm* Pre-test IG 25.7±6.9, Post-test IG 27.2±6.3 Pre-test CG 27.5±6.9, Post-test CG 27.3±6.8
Yahata et al. [65]	*N* = 16	Plantar flexors	Static stretching intervention of 6 × 5 min performed two times per week for 5 weeks using a stretching board	*Maximal isometric strength in the plantar flexors at 30°plantar flexion (in Nm)* Pre-test IG 65.4 ± 15.9, Post-test 66.8 ± 17.6 Pre-test CG 64.2 ± 17.8, Post-test CG 59.4 ± 15.5 *Maximal isometric strength in the plantar flexors in neutral position (in Nm)* Pre-test IG 158.8 ± 31.7, Post-test IG 167.9 ± 33.6 Pre-test CG 151.6 ± 34.9, Post-test CG 151.9 ± 34.1 *Maximal dynamic strength in the plantar flexors at 30°/s (in Nm)* Pre-test IG 134.7 ± 27.5, Post-test IG 144.0 ± 25.4 Pre-test CG 128.0 ± 27.0, Post-test CG 131.1 ± 24.7 *Maximal dynamic strength in the plantar flexors at 120°/s (in Nm)* Pre-test IG 82.9 ± 24.0, Post-test IG 84.2 ± 22.5 Pre-test CG 77.5 ± 20.2, Post-test CG 77.1 ± 17.6 *Muscle thickness in the medial head of the gastrocnemius (in mm)* Pre-test IG 20.2 ± 2.1, Post-test IG 20.1 ± 2.0 Pre-test CG 19.2 ± 3.0, Post-test CG 19.3 ± 2.8 *Muscle thickness in the lateral head of the gastrocnemius (in mm)* Pre-test IG 17.6 ± 2.4, Post-test IG 17.6 ± 2.3 Pre-test CG 16.5 ± 1.7, Post-test CG 16.8 ± 1.6

*Abbreviations* IG = intervention group, CG = control group, n = number of participants, N = Newton, mm = milli meters, kg = kilogram, Nm = Newtonmeter, BW = bodyweight

**Table 2:** Calculated pooled effect sizes with 95% CIs, degrees of freedom, p-values for significance and heterogeneity

Parameter	Effect size	95% CI	p-value	Heterogeneity (τ²)
*Maximum Strength overall*	0.30*	*0.14, 0.46*	*< 0.001*	*0.01*
Maximum Strength < 15 min	0.21	-0.06, 0.44	0.06	0.02
Maximum Strength ≥ 15 min	0.45*	0.29, 0.62	< 0.001	0.0
Maximum Strength < 6w	0.16*	0.05, 0.26	0.006	0.0
Maximum Strength ≥ 6w	0.36*	0.13, 0.59	0.003	0.04
Maximum Strength < 5x	0.26*	0.14, 0.38	< 0.001	0.0
Maximum Strength ≥ 5x	0.32*	0.05, 0.6	0.025	0.04
*Muscle Size overall*	0.2*	*0.008, 0.32*	*0.003*	*0.0*
Muscle Size < 15 min	0.13	-0.05, 0.30	0.14	0.0
Muscle Size ≥ 15 min	0.28*	0.12, 0.44	0.005	0.0
Muscle Size < 6w	-0.05	-0.12, 0.031	0.13	0.0
Muscle Size ≥ 6w	0.26*	0.14, 0.37	< 0.001	0.0
Muscle Size < 5x	0.09	-0.15, 0.33	0.39	0.0
Muscle Size ≥ 5x	0.27*	0.13, 0.42	0.002	0.0

*Abbreviations* 95% CI = 95% Confidence interval, 6w = 6 weeks, 5x = 5 sessions per week, * = significant change

#### *Maximal Strength Capacit*y

Static stretching showed a small positive effect on maximal strength (*d* = 0.30, *p* < 0.001, 95% CI 0.14 to 0.46, τ²=0.01, 36 studies with 85 ES, Table 1). The certainty about the evidence is low. Meta-regression showed stretching duration positively influenced maximal strength (*p* = 0.04, estimate: 0.005), while a tendency was reported for intervention period (*p* = 0.06, estimate: 0.06). No significant result could be found for training frequency (*p* = 0.64).

**Fig. 2:**
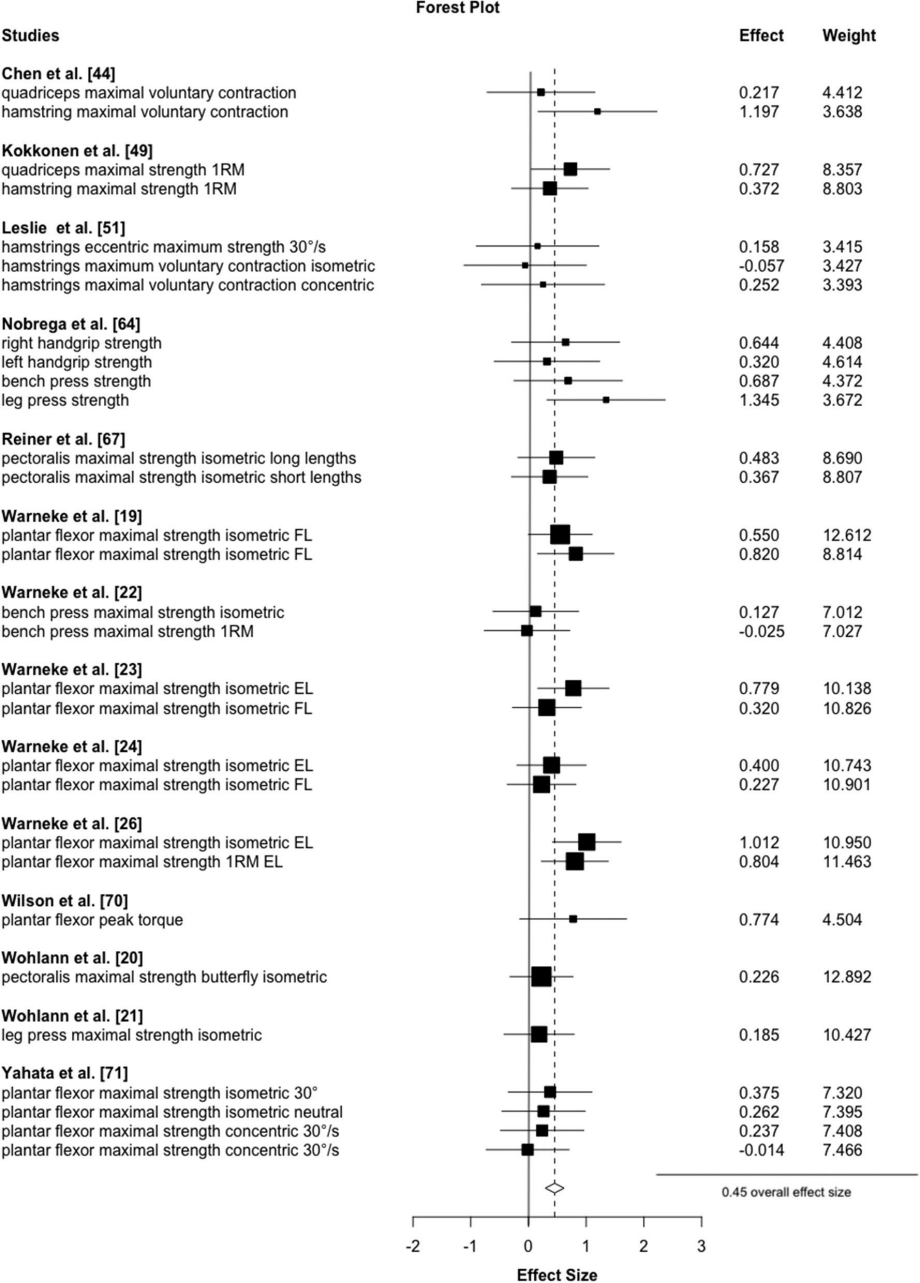
Illustrates the meta-analytical results of long stretching durations. Legend: 1RM = one repetition maximum, EL = extended leg, FL = flexed leg

**Fig. 3:**
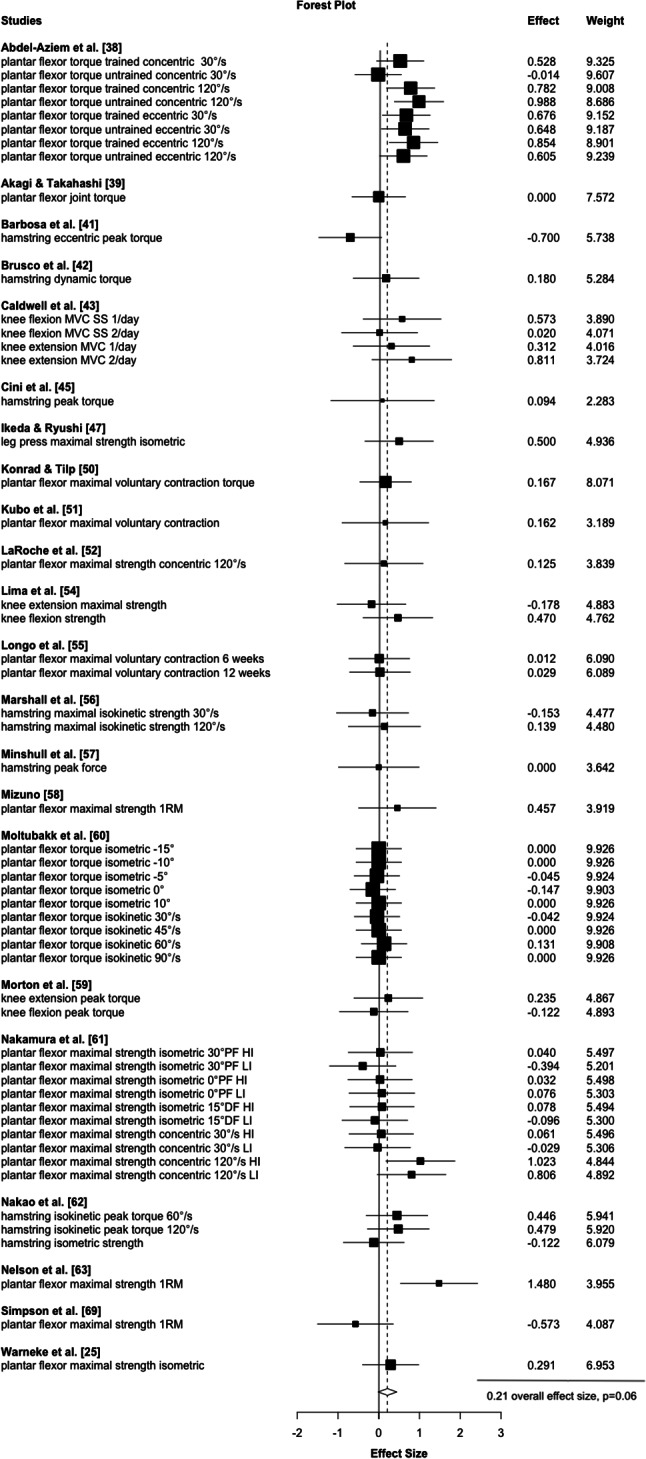
Illustrates the meta-analytical results of short stretching durations. Legend: HI = high intensity group, LI = low intensity group, 1RM = one repetition maximum

Accordingly, higher stretch durations (≥ 15 min) induced small strength increases (*d* = 0.45, *p* < 0.001, 95% CI 0.29 to 0.62, τ²=0.0, 14 studies, 30 ES, Fig. 2) which were opposed to shorter durations (< 15 min) which revealed a small-magnitude, not significant effect (*d* = 0.21, *p* = 0.06, 95% CI -0.06 to 0.44, 22 studies, 55 ES, Fig. 3) with a significant mean ES difference (*p* = 0.01). The certainty about the evidence is moderate.

Similar to stretch duration, longer program durations (> 6 weeks) achieved small strength increases (*d* = 0.36, *p* = 0.003, 95%CI 0.13 to 0.59, τ²=0.04, 24 studies with 51 ES) while shorter durations yielded only trivial improvements (*d* = 0.16, *p* = 0.006, 95%CI 0.05 to 0.26, τ²=0.0, 12 studies, 34 ES), with a significantly higher mean effect for longer intervention periods (*p* = 0.03). The certainty about the evidence is moderate. High training frequencies (more than five stretching sessions per week) led to small-magnitude strength increases (*d* = 0.32, *p* = 0.025, 95% CI 0.05 to 0.6, τ²=0.04, 16 studies, 40 ES). Less than five sessions per week yielded only a small effect size (*d* = 0.26, *p* < 0.001, 95%CI 0.14 to 0.38, τ²=0, 20 studies with 45 ES), without a significant difference in group mean effects (*p* = 0.39). The certainty about the evidence is low.

#### Hypertrophy

**Fig. 4:**
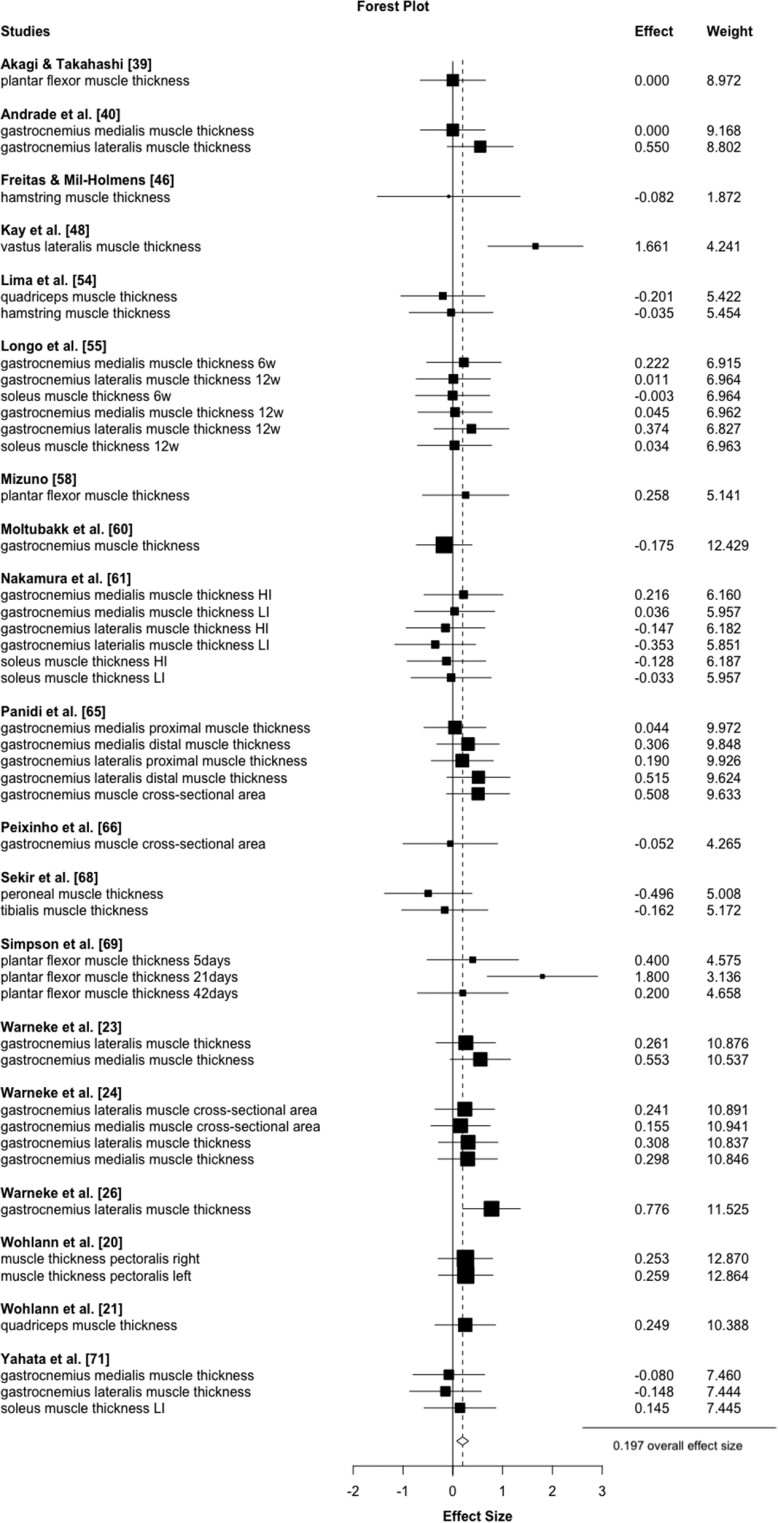
Forest plot for all included studies on stretch-mediated hypertrophy

For hypertrophy, a trivial positive effect of stretching was found (*d* = 0.20, *p* = 0.003, 95% CI 0.08 to 0.32, τ²=0.0, 19 studies, 45 ES) (see Fig. 4). The certainty about the evidence is low. While the meta regression (*p* = 0.23–0.88) revealed no significant influence of any included moderator, long-duration stretching (≥ 15 min) had a small effect size (*d* = 0.28, *p* =  0.005, 95% CI 0.12 to 0.44, τ²=0.0, 7 studies, 17 ES) without a significant difference compared to shorter durations (*p* = 0.29) that, in turn, failed reaching a significant effect (*d* = 0.13, *p* = 0.14, 95%CI -0.05 to 0.30, τ²=0.0, 12 studies with 28 ES). Similarly, studies that performed stretching for more than 6 weeks revealed *d* = 0.26, *p* < 0.001 extracted from 16 studies with 35 ES, while shorter training periods failed to reach the level of significance (*d*= -0.05, *p* = 0.13 from 3 studies and 10 ES) with higher effects for longer periods (*p* = 0.006). If stretching was performed more than 5 times per week, there were significant small magnitude increases in muscle size (*d* = 0.27, *p* = 0.002, from 11 studies with 28 ES), opposed by no significant effect for lower training frequencies (*d* = 0.09, *p* = 0.39), without a significantly higher mean effect size for higher frequencies (*p* = 0.31). The certainty about the evidence is low for all effects.

#### Publication Bias

**Fig. 5:**
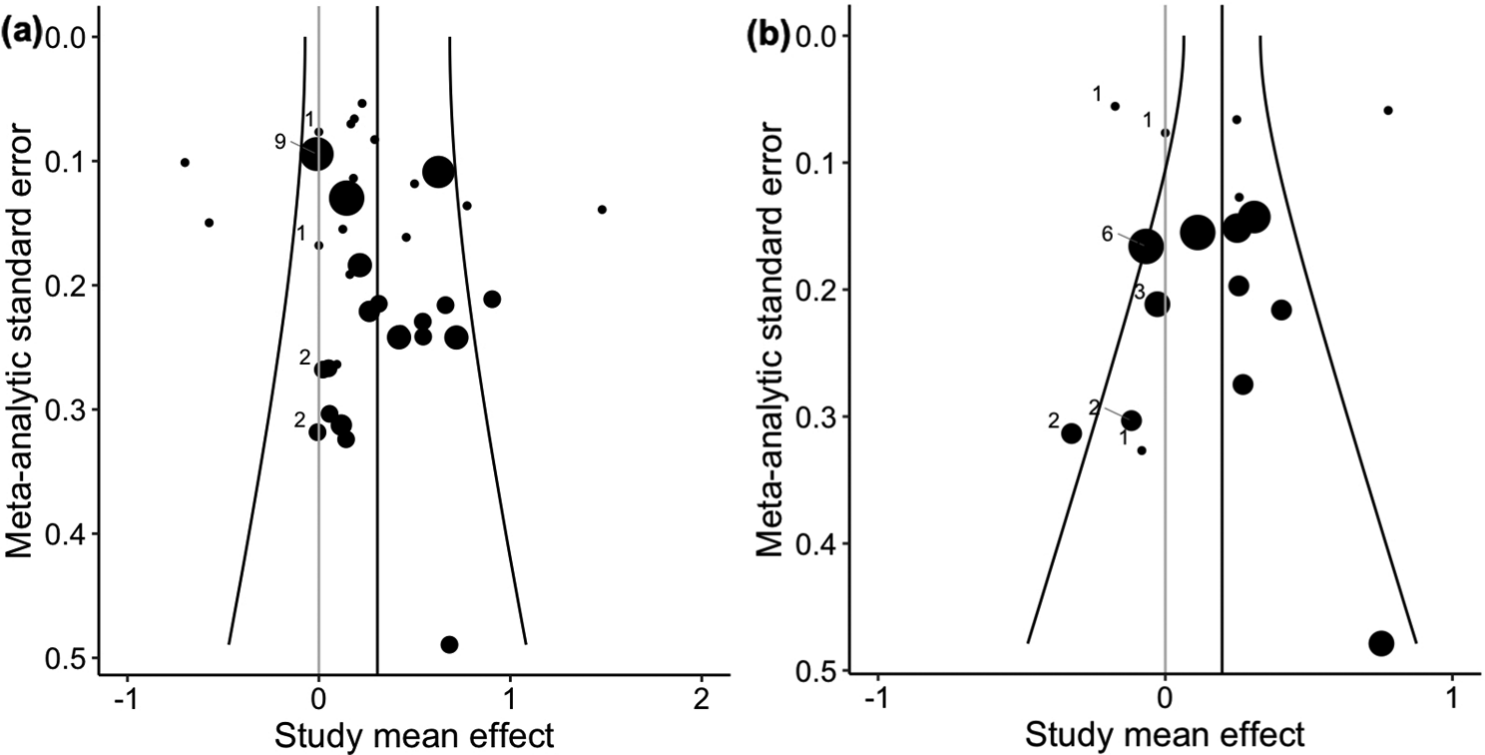
Shows funnel plots for visual publication bias inspection, with (**a**) for maximal strength studies and (**b**) for hypertrophy studies. Plot size illustrates the number of outcomes in the respective study that were pooled and weighted in the meta-analytical calculation

Visual inspection of funnel plots (Fig. 5) revealed no indication of a publication bias for maximal strength as well as for muscle volume. Consistently, for both outcomes, Egger’s regression tests showed no publication bias *p* = 0.23–0.31.

## Discussion

In accordance with previous research, the present systematic review found chronic static stretching to increase (a) maximum strength [11, 12, 17, 18], and (b) muscle size [16]. With stretching duration and a tendency for intervention time as moderating training parameters for maximal strength, our results indicate longer stretching durations to be of superior effectiveness. While overall stretch-induced hypertrophy showed small effects (d = 0.2), these effects seem attributable to stretching durations of ≥ 15 min, intervention periods of > 6 weeks and training frequencies of ≥ 5 times as lower dosage did not reach the level of significance in subgroup calculations (*p* = 0.14–0.39). The possible necessity of high stretching volumes with regard to improvements in strength and muscle volume is in line with results from animal studies [73, 74].

As pointed out, early evidence had mostly suggested that stretching does not modify morphological and functional muscle parameters in humans [11, 12, 15]. However, this assumption was based on a lack of studies using high to very high stretch durations. Even the most recent review of Arntz et al. [18] did not include long duration studies [19–21, 25, 26, 75, 76], while Panidi et al. [16] included only one long-duration study [26]. Since animal research indicated a potential dose-reponse relationship [14, 77], a meta-regression was performed that confirmed stretching duration to significantly moderate strength adaptations. While in contrast, the regression did not reveal such a relationship for muscle hypertrophy, significant muscle size enhancements were only obtained in higher dosage in subgroup analyses (≥ 15 min stretching, ≥6 weeks intervention period, ≥5x stretching per week). Compared to animals with reported muscle mass increases of up to 300% [78], human hypertrophy effects must be considered small. These differences could be attributed to diverse factors. Compared to animals, human muscle protein synthesis is slower [79–81]. This may be one explanation for a lack of hypertrophy in response to 30 min of stretch reported by Yahata [65]. Nevertheless, by using stretching durations of accumulated 15 min per session, Wohlann et al. [20] obtained significant muscle hypertrophy. There were differences in the intervened muscle groups, Wohlann used 4x weekly pectoralis stretching, while calf muscle stretching performed by Yahata and colleagues [65] was applied only twice per week. The potential role of training frequency is supported by consistent hypertrophy effects in all Warneke et al. studies [23, 24, 26], who used daily stretching. The results of the meta-analysis partly confirm this assumption, although meta regression did not reach the level of significance for both, maximal strength and hypertrophy. However, subgroup analysis for hypertrophy showed only more frequent training application to produce significant effects, while no significant influence of frequency was observed for strength increases.

Several mechanisms could explain the stretch-induced increases in muscle size or strength. First and foremost, it may be speculated that time under tension is not only paramount for gains in muscle volume following resistance training [82] but also following stretching [83], which would be in agreement with our results, showing the stretching duration to be important for strength (meta regression: *p* = 0.038), but also for hypertrophy, as only with ≥ 15 min muscle size did increases occur. Accordingly, the literature shows high mechanical tension imposed on the sarcomere could trigger protein synthesis [84, 85]. In quails and chickens, progressive stretching induced fast hypertrophy alongside serial sarcomereogenesis during the first days of the intervention [78]. However, when the stretching stimulus remained unmodified during such a program, initial increases in muscle cross-sectional area started to disappear [86]. Ashmore [87] suggested that the mechanical tension caused by stretching would lead to high stresses and compensatory adaptations in the sarcomere. It has, furthermore, been hypothesized that an increased total amount of sarcomeres reduces tension and with this stress on the individual sarcomere [86]. Thus, to increase training intensity and to ensure continuously strong tensioning of the sarcomere, the stretching stimulus needs to be re-adjusted. Indeed, Antonio & Gonyea [78] achieved the highest gains in muscle mass and hypertrophy by increasing the stretch intensity, starting with 10% of the body weight up to 35% after 5 weeks of chronic stretch in quails.

Another theory postulates that chronic stretch creates hypoxic conditions which are similar to those during blood flow restriction. Reducing arterial perfusion has been demonstrated to increase lactate levels, growth hormone concentrations, and inflammatory cytokines such as interleukin-6 [88, 89]. Such metabolic milieu may represent a potent stimulus for mTOR signaling [90–92]. Interestingly, Jessee et al. [93] showed that blood flow restriction induces hypertrophy, however, it seems of minor relevance for maximum strength increases. Hotta et al. [94] observed acute decreases of blood flow during 30 min of stretching in animals. Studies measuring the metabolic muscle response to stretching would thus be warranted in order to further delineate the potential relevance of the abovementioned factors.

In sum, irrespective of initial processes, muscle hypertrophy requires an increase in muscle protein synthesis. Suzuki & Takeda [95] and Kremer [96] described the activation of stretch-activated channels and thus, the stimulation of the mTOR/p70S6K/PI3K pathway [97–99]. The literature emphasizes the importance of mechanical tension (e.g., through stretching) to trigger anabolic signaling pathways, with the stimulation of protein synthesis [100–103] as an underlying mechanism of hypertrophy (and maximal strength) [104–106]. Van der Pjil et al. [107, 108] indicated the relevance of titin unfolding in hypertrophy (in parallel and longitudinal), supporting the hypothesis of high intensities [109]. Conversely, Fowles et al. [110] were not able to show acute increases in protein synthesis after 33-minutes of stretching in humans, although significant increases in protein synthesis rates had been reported in animals [100, 102, 103, 111]. The stronger response in animals could hence be explained by a higher protein synthesis rate [80, 81].

With regard to the increases in maximum strength, it may be expected that the increases in muscle volume would drive the strength gains. This would require hypertrophy to precede enhanced strength. However, no study has investigated the temporal association of both factors. In addition, effect sizes were trivial to small for muscle volume but moderate for strength. Another theory may attribute the improvements to neural adaptations [112, 113]. The studies by Warneke et al. [19, 26] and Nelson et al. [60], on the one hand, provide support for this assumption as they detected strength increases in the non-stretched contralateral leg. However, on the other hand, Holly et al. [114] and Barnett et al. [115] showed no significant increase in EMG activity during stretching in animals. Furthermore, Sola et al. [116] found stretch-mediated hypertrophy in denervated muscles, indicating a minor role of neural aspects. Therefore, to clarify the role of neural aspects in stretch-mediated adaptations, further research seems necessary.

Even though muscle hypertrophy only occurs using higher dosage stretching, our work has significant clinical implications. In general, stretching may represent an alternative to conventional resistance training interventions inducing muscle size- and strength increases. Nevertheless, several aspects must be considered. While Currier et al. [117] showed moderate to large magnitude maximal strength and muscle size increases of ES = 0.51 and ES = 1.60, respectively, when using resistance training, the present study’s small magnitude effect sizes of ES = 0.28 and ES = 0.45, respectively, showed that even long stretching durations were less effective. Assuming about one hour of stretching on one isolated muscle to achieve meaningful muscle hypertrophy [83] seems, on the one hand, of limited practical relevance [85]. On the other hand, passively induced mechanical tension via stretch training could be included into daily life, with for example using splints/ortheses during sitting in the office or while watching television [118]. A further benefit might be the potential applicability for people lacking motivation or ability to perform resistance training (e.g., patients with unstable cardiovascular diseases), if heavy resistance training is contraindicated, or after muscle, ligament or bone injuries leading to prolonged times of immobilization. Thus, (probably only) for conditioned populations, stretching could provide a sufficient alternative, especially since no training supervision is necessary to ensure safe exercise execution. Although stretching could be a valuable training intervention, it should only temporarily substitute or, even better, supplement classical training regimes. This is of importance because although stretching has been shown to be beneficial for cardiovascular health [119], it may not add as efficiently to the recommended levels of physical activity (e.g. by the World Health Organization, 150 min of moderate or 75 min of vigorous activity per week) as other activities such as walking, running, team sports, or resistance training.

Several aspects call for further research. Even though significant stretch-induced muscle hypertrophy in response to stretching durations of ≥ 15 min was identified, this was based on only 7 studies with a range of 3 × 5 min to one hour of stretching, with the highest effects originating from one research group [19–21, 23–26, 76]. Thus, further studies are requested to confirm or disconfirm the results. Furthermore, all long-lasting stretch interventions (more than one hour) were performed with high stretching frequency and intervention periods (≥ 6 weeks), increases in maximal strength and muscle volume cannot be clearly ascribed to one of these parameters. Further studies should hence examine long-lasting stretch interventions of < 6 weeks and/or ≤ 5 sessions per week. Moreover, the role of stretch intensity merits further investigation. Reporting stretch intensity using individual pain perception seems of questionable validity [120]. However, it is well known from strength training that training intensity seems to be of crucial importance for adaptations, especially with regard to maximum strength increases [121]. Considering the importance of titin unfolding, which is assumed to occur exclusively in maximally stretched sarcomeres, reaching high degrees of stretch could be hypothesized to be of paramount importance [109, 122].

Despite some plausible theories [83], the underlying mechanisms remain speculative. While many physiological parameters were assessed in animals, no studies examined signaling pathways and possible alterations of protein synthesis in humans. Furthermore, research has almost exclusively focused on skeletal muscle. Interestingly, it has been shown that the connective tissue can exert significant force transmission effects [123]. Therefore, it may be prudent for future trials to consider multiple tissues.

Some increases in the examined parameters were surprisingly high in studies included in our review. Nelson et al. [60] reported an improvement in maximal strength of 29% (d = 1.48) in the stretched leg and a gain of about 11% (d = 0.46) in the contralateral control leg following 4 × 30 s stretching three times per week for ten weeks. Mizuno [55] found increases of 24% using static stretching three times per week for eight weeks, while Panidi et al. [69] detected hypertrophy effects of up to 23%. When these short duration stretching results are compared to those from strength training [124], the listed stretch-induced adaptations seem unreasonably high, even though participants are partially classified untrained to recreationally active. Against this background, it will be of interest to further identify moderator variables determining strong and weak stretch responders.

Lastly, testing for significant differences of mean effects to provide a valuable statement of subgroup differences was performed using the Welsh test. This testing procedure must be considered a supplementation of the main statistics and must be interpreted with caution, as no specific pooling for dependent outcomes was possible. If one study provided multiple outcomes, effect size means were calculated, meaning each study corresponded to one outcome, which reduced this limitation.

## Conclusions

The present systematic review provides low- to moderate-certainty evidence that chronic static stretching increases maximum strength and muscle size. While the overall effects are small if existent, comparatively high effort seems necessary with longer stretching- and intervention periods (≥ 15 min, ≥ 6 weeks) and greater frequencies (≥ 5x/week) seem particularly effective. The exact physiological mechanisms causing potential effects remain a matter of debate. Nevertheless, even though less effective compared to resistance training, high volume stretching might provide a valuable alternative under special circumstances, e.g., if traditional resistance training is contraindicated.

### Electronic Supplementary Material

Below is the link to the electronic supplementary material.

Supplementary Material 1

## Data Availability

Data can be provided on reasonable request. Supplemental materal associated with this article can be found in the online version.

## Abbreviations

CI: confidence interval
ES: effect size
M: mean
SD: standard deviation
SMD: standardized mean differences
