# Supplementary material for: Effects of Chronic Static Stretching on Maximal Strength and Muscle Hypertrophy: A Systematic Review and Meta-Analysis with Meta-Regression
Source: Sports Med Open. 2024 Apr 19;10:45. doi: 10.1186/s40798-024-00706-8 (PMC11026323; doi:10.1186/s40798-024-00706-8)
Supplement: Supplementary file 1 — Supplementary Material 1 [file 40798_2024_706_MOESM1_ESM.docx]

| Authors | 2. | 3. | 4. | 5. | 6. | 7. | 8. | 9. | 10. | 11. | Score |
| --- | --- | --- | --- | --- | --- | --- | --- | --- | --- | --- | --- |
| Abdel-Aziem & Mohammad 2012 | N | N | N | N | N | N | N | N | Y | Y | 2/10 |
| Akagi & Takahashi 2014 | Y | N | Y | N | N | N | N | N | Y | Y | 4/10 |
| Andrade et al. 2020 | Y | N | N | N | N | N | Y | Y | Y | Y | 5/10 |
| Barbosa et al. 2020 | Y | Y | Y | N | N | Y | Y | Y | Y | Y | 8/10 |
| Brusco et al. 2019 | N | N | Y | N | N | N | Y | N | Y | Y | 4/10 |
| Caldwell et al. 2019 | Y | N | N | N | N | N | N | N | Y | Y | 3/10 |
| Chen et al. 2011 | N | N | Y | N | N | N | N | N | Y | Y | 3/10 |
| Cini et al. 2020 | Y | Y | Y | N | N | Y | Y | Y | Y | Y | 8/10 |
| Freitas & Mil-Homens 2015 | Y | N | N | N | N | Y | N | N | Y | N | 4/10 |
| Ikeda & Ryushi 2018 | Y | N | Y | N | N | N | N | N | Y | Y | 4/10 |
| Kay et al. 2018 | Y | N | Y | N | N | N | N | N | Y | Y | 4/10 |
| Kokkonen et al. 2007 | Y | N | N | N | N | N | Y | N | Y | Y | 4/10 |
| Konrad & Tilp 2014 | Y | N | N | N | N | N | N | N | Y | Y | 3/10 |
| Kubo et al. 2002 | Y | N | Y | N | N | N | N | N | Y | Y | 4/10 |
| LaRoche et al. 2008 | Y | N | Y | N | N | N | N | N | Y | Y | 4/10 |
| Leslie et al. 2017 | Y | N | N | N | N | N | Y | N | Y | Y | 4/10 |
| Lima et al. 2015 | Y | N | Y | N | N | N | N | N | Y | Y | 4/10 |
| Longo et al. 2021 | Y | N | Y | N | N | N | Y | N | Y | Y | 5/10 |
| Marshall et al. 2011 | Y | N | Y | N | N | N | N | N | Y | Y | 4/10 |
| Minshull et al. 2013 | Y | N | N | N | N | N | N | N | Y | Y | 3/10 |
| Mizuno 2019 | N | N | N | N | N | N | N | N | Y | Y | 2/10 |
| Motulbakk et al. 2021 | Y | N | Y | N | N | Y | N | N | Y | Y | 5/10 |
| Morton et al. 2011 | Y | Y | N | N | N | N | Y | N | Y | Y | 5/10 |
| Nakamura et al., 2021 | Y | N | Y | N | N | N | N | N | Y | Y | 4/10 |
| Nakao et al. 2019 | Y | N | Y | N | N | N | Y | Y | Y | Y | 6/10 |
| Nelson et al. 2012 | Y | N | N | N | N | N | N | N | Y | Y | 3/10 |
| Nobrega et al. 2005 | N | N | N | N | N | N | N | N | Y | Y | 2/10 |
| Panidi et al. 2021 | Y | N | Y | N | N | Y | N | N | Y | Y | 5/10 |
| Peixinho et al. 2021 | Y | N | Y | N | N | N | Y | N | Y | Y | 5/10 |
| Reiner et al. 2023 | Y | N | Y | N | N | N | N | N | Y | Y | 4/10 |
| Sekir et al. 2017 | Y | Y | Y | N | N | Y | Y | Y | Y | Y | 8/10 |
| Simpson et al. 2017 | Y | N | N | N | N | Y | N | N | Y | Y | 4/10 |
| Warneke et al. 2022a | N | N | Y | N | N | N | N | N | Y | Y | 3/10 |
| Warneke et al. 2022b | N | N | Y | N | N | N | N | N | Y | Y | 3/10 |
| Warneke et al. 2022c | N | N | Y | N | N | N | N | N | Y | Y | 3/10 |
| Warneke et al. 2023a | Y | N | Y | N | N | N | N | N | Y | Y | 4/10 |
| Warneke et al. 2023b | Y | N | Y | N | N | N | N | N | Y | Y | 4/10 |
| Warneke et al. 2023c | Y | N | Y | N | N | N | N | N | Y | Y | 4/10 |
| Wilson et al. 2019 | Y | N | N | N | N | N | Y | Y | Y | Y | 5/10 |
| Wohlann et al. 2023 | N | N | Y | N | N | N | N | N | Y | Y | 3/10 |
| Wohlann et al. 2024 | Y | N | Y | N | N | N | N | N | Y | Y | 4/10 |
| Yahata et al. 2021 | N | N | Y | N | N | N | Y | Y | Y | Y | 5/10 |

Table A PEDro Scale based quality assessment of the included studies
